# Intra-abdominal infection combined with intra-abdominal hypertension aggravates the intestinal mucosal barrier dysfunction†

**DOI:** 10.1042/BSR20170931

**Published:** 2018-01-10

**Authors:** Yuan Li, Jianan Ren, Xiuwen Wu, Jieshou Li

**Affiliations:** Department of General Surgery, Jinling Hospital, Medical School of Nanjing University, Nanjing 210002, China

**Keywords:** Abdominal Compartment Syndrome, Intra-Abdominal Infection, Intra-Abdominal Hypertension, Intestinal Mucosal Barrier, Intestinal Permeability

## Abstract

Some patients with intra-abdominal infection (IAI) may develop intra-abdominal hypertension (IAH) during treatment. The present study investigated the impact of IAI combined with IAH on the intestinal mucosal barrier in a rabbit model. Forty-eight New Zealand white rabbits were randomly divided into four groups: (i) IAI and IAH; (ii) IAI alone; (iii) IAH alone; and (iv) Control group. IAI model: cecal ligation and puncture for 48 h; IAH model: raised intra-abdominal pressure (IAP) of 20 mmHg for 4 h. Pathological changes in intestinal mucosa were confirmed by light and scanning electron microscopy. FITC-conjugated dextran (FITC-dextran) by gavage was used to measure intestinal mucosal permeability in plasma. Endotoxin, d-Lactate, and diamine oxidase (DAO) in plasma were measured to determine intestinal mucosal damage. Malonaldehyde (MDA), superoxide dismutase (SOD), and GSH in ileum tissues were measured to evaluate intestinal mucosal oxidation and reducing state. Histopathologic scores were significantly higher in the IAI and IAH group, followed by IAI alone, IAH alone, and the control group. FITC-dextran, d-Lactate, DAO, and endotoxin in plasma and MDA in ileum tissues had similar trends. GSH and SOD were significantly lowest the in IAI and IAH group. Occludin levels were lowest in the ileums of the IAI and IAH group. All differences were statistically significant (*P*-values <0.001). IAI combined with IAH aggravates damage of the intestinal mucosal barrier in a rabbit model. The combined effects were significantly more severe compared with a single factor. IAI combined with IAH should be prevented and treated effectively.

## Introduction

Intra-abdominal infection (IAI), including diffuse peritonitis and abdominal abscess, has the second highest incidence [1] of infectious diseases amongst inpatients. Despite diagnostic and therapeutic advances over the past decades, the mortality and complication rates of IAI remain high [2].

Intra-abdominal hypertension (IAH) is increasingly acknowledged as a difficult critical illness in clinical practice. The definition of IAH [3] is a sustained or repeated pathological elevation in intra-abdominal pressure (IAP) ≥12 mmHg. Because the rate of IAH morbidity and mortality remains high, it is still an enormous challenge for physicians [3].

For patients who have undergone major abdominal surgery or who have severe abdominal trauma, severe burns, or severe acute pancreatitis, IAI may coexist with IAH [4]. If treatment is not given promptly or is incorrect, patients can develop multiple organ dysfunction syndrome (MODS).

The gut is considered the motor of MODS and has a central role in disease progression [5]. Damage of the intestinal mucosal barrier function is an important factor of MODS [6]. Previous studies demonstrated that IAI [7] or IAH [8] alone caused intestinal mucosal barrier dysfunction. However, no studies have focussed on damage of intestinal mucosal barrier function when IAI coexists with IAH. The underlying pathophysiological changes of the intestinal mucosal barrier function during IAI combined with IAH are still unclear.

Therefore, we established a novel rabbit model of IAI combined with IAH. We examined pathological changes of the intestinal mucosa, intestinal mucosa barrier function, and intestinal mucosa redox status amongst disparate rabbit models (IAI alone, IAH alone, IAI combined with IAH, and control), and explored the effect of IAI combined with IAH on intestinal mucosa barrier function.

## Methods

### Animals

Forty-eight New Zealand white rabbits aged 6–8 months, weighing 2.50 ± 0.22 kg, were provided by Jinling Rabbit Breeding Farm (Nanjing, China). All rabbits were used and kept in a clean laboratory in Jinling Hospital (Nanjing, China). All the experimental protocols were approved by the Animal Ethics Committee of Jinling Hospital.

The rabbits were randomly divided into four groups: (i) IAI and IAH; (ii) IAI alone; (iii) IAH alone; and (iv) Control group. All the experiments were conducted in sodium pentobarbital-anesthetized (30 mg/kg, via an ear vein) rabbits. All the rabbits were fasted but had free access to water after experimental or sham operation.

### Rabbit model of IAI alone

The IAI rabbit model was established by the cecal ligation puncture (CLP) procedure. Lower quadrants of the abdomen were shaved with an electric razor and sterilized with 75% ethanol. The contents were pushed gently toward the distal cecum. The cecum was perforated 2 cm away from the appendix and ligated at the proximal cecum. A small amount of feces was pressed from the site of perforation to ensure the puncture, and saline was injected intraperitoneally (37°C; 5 ml per 100 g body weight) and abdominal closure was performed. Then, rabbits were monitored for 48 h to observe adverse outcomes. The center portion of the abdominal wall was penetrated with an intravenous catheter (8Fr, SCW Medicath Ltd., Shenzhen, China) and the catheter was sutured on to the skin. The distal end of the catheter was connected to an IAP monitor (KrosFlo®, U.S.A.) and the other end was closed for 4 h.

### Rabbit model of IAH alone

The IAH rabbit model was established by nitrogen pneumoperitoneum. Lower quadrants of the abdomen were shaved with an electric razor and sterilized with 75% ethanol. Sham operations were performed after waiting for 48 h. The rabbits were supine on a restraining device with an electric heating pad to maintain body temperature at 37°C during the procedure. The center portion of the abdominal wall was penetrated with an intravenous catheter and the catheter was sutured on to the skin. The distal end of the catheter was connected to an IAP monitor, and the other end was connected to a nitrogen supply. After the target IAP (20 mmHg) was achieved, a low flow of nitrogen was used for maintenance for 4 h.

### Rabbit model of IAI combined with IAH

The CLP procedure (as described in the Rabbit model of IAI alone) was performed after anesthesia (waiting for 48 h). Nitrogen pneumoperitoneum was established for 4 hours at an IAP of 20 mmHg (as described in Rabbit model of IAH alone). The control group underwent sham CLP operations (waiting for 48 h) and sham nitrogen pneumoperitoneum (waiting for 4 h).

### Samples collection and treatment

Portal vein blood (4 ml) and intestinal mucosa tissue (2 g) were collected after the model was established. Portal vein blood mixed with anticoagulation agents was divided into four parts and stored at 4°C in the dark. Intestinal mucosa tissues were divided into three parts. Two parts were directly stored in liquid nitrogen. The other part was used to prepare tissue homogenates in an ice bath. Supernatant was extracted by centrifugation (3000 rpm, 4°C, 10 min) and stored in liquid nitrogen.

### Evaluation of intestinal mucosal injury

#### Pathological changes observed by light and transmission electron microscopy

One part of the mucosa tissue sample was paraformaldehyde fixed for 24 h, embedded in paraffin, sectioned, and stained with Hematoxylin-Eosin for light microscopy. Another part was fixed in 0.5% glutaraldehyde, embedded in paraffin, and sectioned for TEM.

Histopathologic scores were graded under light microscopy, including the extent of lesions, infiltration depth of lesions, edema, inflammatory cell infiltration, and other lesions (assessment scores range from 0 to 4, respectively). The mean of the total scores was the histopathologic score.

#### Intestinal mucosa barrier function

Intestinal mucosa barrier permeability was measured by FITC-conjugated dextran (FITC-dextran, 4 kDa, Sigma–Aldrich®, St. Louis, U.S.A.). FITC-dextran (10 mg/kg) was administered through a gastric tube (6#, Jiangyang®, Yangzhou, China), 30 min before the nitrogen pneumoperitoneum process ended or the sham nitrogen pneumoperitoneum process ended. The plasma concentration of FITC-dextran was tested by fluorospectrophotometer (F93, Shanghai Precision & Scientific Instrument Co. Ltd., China).

The plasma concentrations of endotoxin (LPS), d-Lactate, and diamine oxidase (DAO) were measured by ELISA kits (Jiancheng Bioengineering Institute, Nanjing, China) according to the manufacturer’s instructions. Each experiment was repeated three times.

#### The expression of occludin in intestinal mucosa tissues

Intestinal mucosa tissue supernatant was investigated by Western blot to determine the level of occludin and the mRNA expression of occludin was determined by quantitative real-time PCR (real-time PCR).

#### Western blotting

Forty micrograms of total protein extracts were separated by SDS/PAGE (AS1012; ASPEN, Wuhan, China) and transferred on to PVDF membranes (IPVH00010; Millipore, Darmstadt, Germany). The membranes were blocked with milk for 1 h at room temperature and incubated in primary antibodies overnight at 4°C. After washing three times with TBS-Tween 20 (TBST), the corresponding secondary antibodies were added and incubated at 37°C for 2 h. Anti-occludin antibody (ab168986) and GAPDH antibody were from Abcam (Cambridge, U.K.). The band density was measured with an image analysis system (AlphaEaseFC, Alpha Innotech, San Jose, U.S.A.).

#### Real-time PCR validation

RNA TRIzol reagent was used to extract total RNA (Invitrogen, Massachusetts, U.S.A.), according to the manufacturer’s instructions. Real-time PCR was performed by using SYBR® Premix ExTaq™ to analyze *occludin* mRNA (Takara, Dalian, China) using a StepOne™ Real-Time PCR machine (Life Technologies, Massachusetts, U.S.A.). Gene expression data were normalized to *GAPDH* mRNA levels. The primer sequences are listed in Table 1.

**Table 1:** Primer sequence for real-time PCR

Target gene	Primer sequence		Product size (bp)
*Occludin*	Forward	5′-GAGACACCTTCCAAAAGGCC-3′	141
	Reverse	5′-CGGATACTCCCTGATCCAGTC-3′	

#### Intestinal mucosa redox status

The concentrations of malonaldehyde (MDA), superoxide dismutase (SOD), and GSH in ileum mucosa tissue supernatants were measured by ELISA kits (Jiancheng Bioengineering Institute, Nanjing, China) according to the manufacturer’s instructions.

### Statistics

Statistical analysis and figure drawings were performed with Prism (GraphPad Software, Inc., version 6.0c). Categorical variables, such as mortality, were analyzed by Fisher’s exact test. Continuous variables are shown as the mean ± S.D. Student’s unpaired *t* test was used to compare continuous variables between groups. *P*-values <0.001 was regarded as statistically different and indicated by *** in figures. All *P*-values were two-sided.

### Ethics approval and consent to participate

All the experimental protocols were approved by Animal Ethics Committee of Jinling Hospital.

## Results

### Mortality

The mortality of the four groups was different. The mortality was highest in the IAI and IAH group (41.7%), followed by the IAH (25.0%), IAI (16.7%), and control groups (0).

### Pathological changes observed by light and transmission electron microscopy

**Figure 1:**
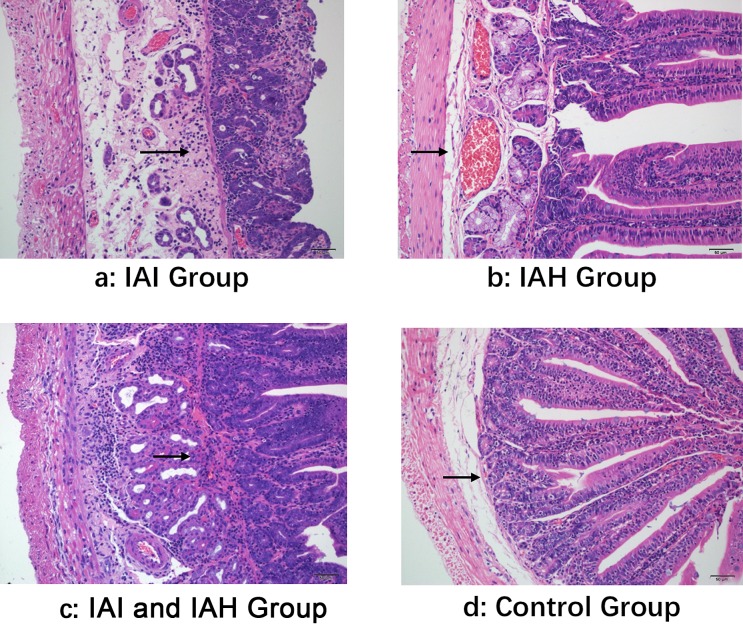
Comparisons of pathological changes of mouse ileal tissues by light microscopy Ileum sections were stained with Hematoxylin-Eosin. Obvious intestinal mucosal damage and normal intestinal mucosal are shown by a black arrow. (**a**) IAI group; (**b**) IAH group; (**c**) IAI and IAH group; (**d**) Control group. Scale bars =50 μm.

As shown in Figure 1, obvious pathological changes were observed under light microscopy and fluorescent microscopy. Intestinal mucosa damage was most severe in the IAI and IAH group, and normal intestinal mucosa was observed in the control group.

Histopathologic scores were assessed in each group. The scores were highest in the IAI and IAH group (7.75), followed by the IAI (5), IAH (4.5), and control groups (0).

**Figure 2:**
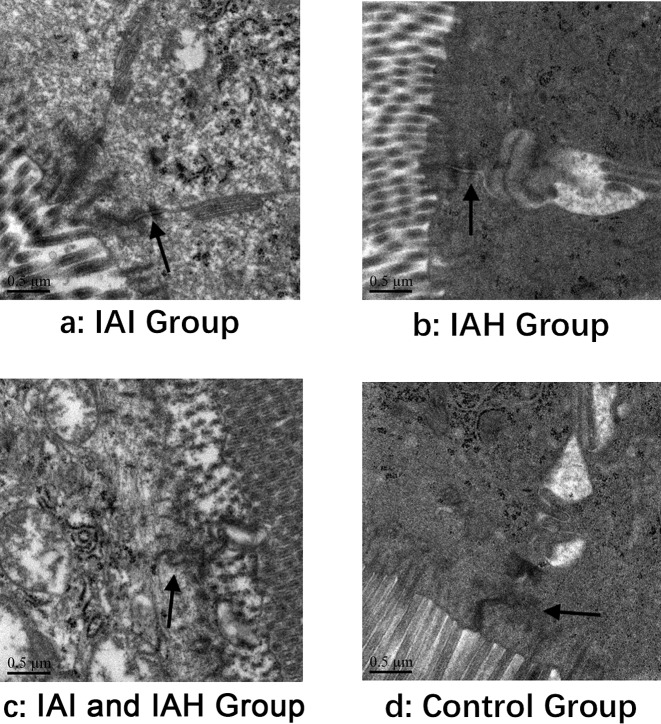
Comparisons of pathological changes of mouse ileal tissues by TEM The deterioration of tight junction and normal structure are indicated by a black arrow. (**a**) IAI group; (**b**) IAH group; (**c**) IAI and IAH group; (**d**) Control group. Scale bars =0.5 μm.

The structural damage of tight junctions was observed by TEM in three experimental groups (Figure 2).

### Intestinal mucosa barrier function

#### FITC-dextran

**Figure 3:**
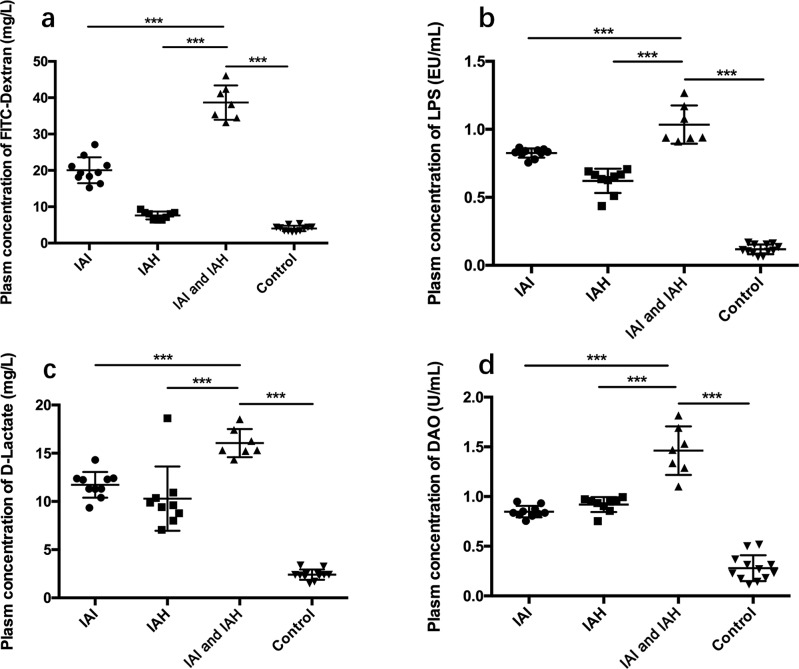
Intestinal mucosa barrier function assessed by the plasma concentrations of FITC-dextran, LPS, d-Lactate, and DAO The data were analyzed by Student’s *t* test and presented as the mean ± S.D. of three independent experiments. Plasma concentrations of (**a**) FITC-dextran; (**b**) LPS; (**c**) d-Lactate; and (**d**) DAO. Values are the means ± S.D. represented by vertical bars. ***P<0.001 compared with control groups.

As shown in Figure 3a, the plasma concentration of FITC-dextran was highest in the IAI and IAH group (38.67 ± 1.78 mg/l, *n*=7), which was significantly higher compared with the IAI (20.06 ± 1.13 mg/l, *n*=10), IAH (7.60 ± 0.36 mg/l, *n*=9), and control groups (4.01 ± 0.22 mg/l, *n*=12).

#### LPS

Similar findings were observed for the plasma concentrations of LPS (Figure 3b), which were highest in the IAI and IAH group (1.035 ± 0.053 EU/ml, *n*=7), which was significantly higher compared with the IAI (0.825 ± 0.011 EU/ml, *n*=10), IAH (0.621 ± 0.030 EU/ml, *n*=9), and control groups (0.118 ± 0.010 EU/ml, *n*=12).

#### d-Lactate

The plasma concentrations of d-Lactate were highest in the IAI and IAH group (16.06 ± 0.55 mg/l, *n*=7), and were significantly higher compared with the IAI (11.73 ± 0.42 mg/l, *n*=10), IAH (10.29 ± 1.11 mg/l, *n*=9), and control groups (2.41 ± 0.15 mg/l, *n*=12), as shown in Figure 3c.

#### DAO

The plasma concentrations of DAO (Figure 3d) were highest in the IAI and IAH group (1.462 ± 0.092 U/ml, *n*=7), and were significantly higher compared with the IAI (0.848 ± 0.018 U/ml, *n*=10), IAH (0.920 ± 0.025 U/ml, *n*=9), and control groups (0.279 ± 0.038 U/ml, *n*=12). All of the above comparisons were statistically significant (*P*<0.001).

#### The tight junction protein: occludin expression was reduced

**Figure 4:**
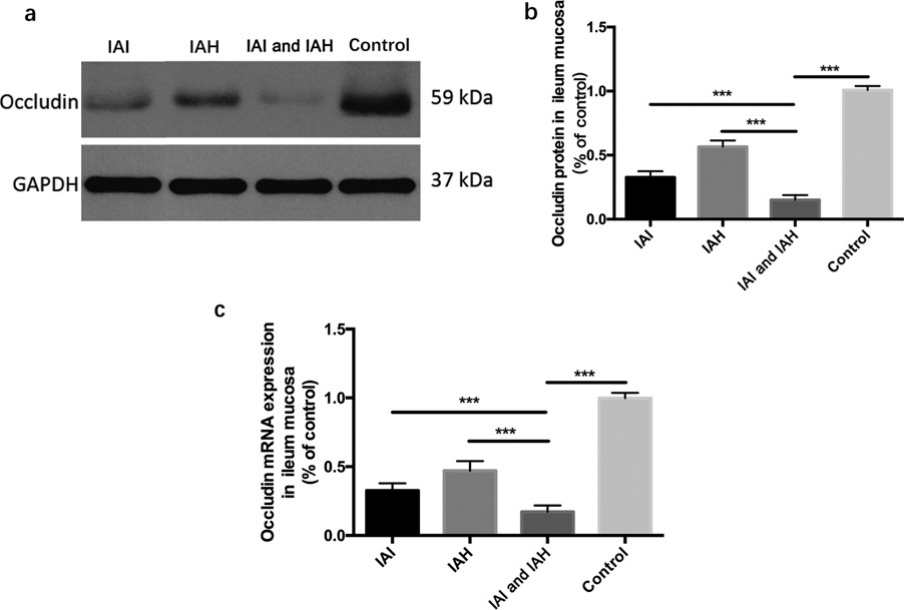
*Occludin* mRNA and protein expressions in mouse ileum mucosa (**a**) The protein expression of occludin was determined by Western blotting. (**b**) The gray values of occludin protein. (**c**) *Occludin* mRNA relative expression level detected by real time-PCR. ***P<0.001 compared with control groups.

Western blot analysis (Figure 4a,b) showed that the expression of occludin protein in ileum mucosa tissue was lowest in the IAI and IAH group compared with the other groups. Occludin expression was lower in the IAI and IAH groups compared with the control group.

Real-time PCR analysis (Figure 4c) revealed that *occludin* mRNA was lowest in the ileum mucosa tissue of the IAI and IAH group, which was significantly lower compared with the IAI, IAH and control groups. All the above comparisons were statistically significant (*P*<0.001).

#### Intestinal mucosa redox status

**Figure 5:**
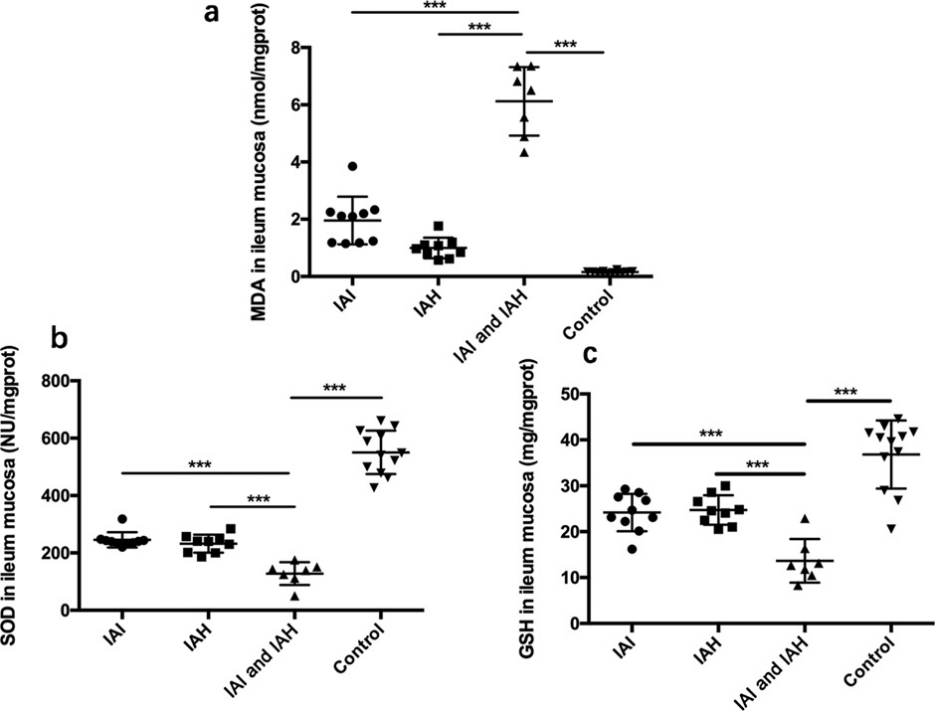
Intestinal mucosa redox status as assessed by the concentrations of MDA, SOD, and GSH in mouse ileum mucosa The data were analyzed by Student’s *t* test and presented as the mean ± S.D. of three independent experiments. Concentrations of (**a**) MDA; (**b**) SOD; and (**c**) GSH in ileum mucosa tissues. Values are the means ± S.D. represented by vertical bars. ***P<0.001 compared with control groups.

As shown in Figure 5a, the concentrations of MDA in the ileum mucosa tissue were highest in the IAI and IAH group (6.12 ± 0.45 nmol/mg prot, *n*=7), and were significantly higher compared with the IAI (1.96 ± 0.26 nmol/mg prot, *n*=10), IAH (0.99 ± 0.120 nmol/mg prot, *n*=9), and control groups (0.15 ± 0.01 nmol/mg prot, *n*=12).

For reducing substances, SOD (Figure 5b) and GSH (Figure 5c) exhibited an opposite trend in ileum mucosa tissues. The lowest levels of SOD and GSH were in the IAI and IAH group (SOD: 128.00 ± 15.07 NU/mg prot; GSH: 13.7 ± 1.80, *n*=7), and these were significantly lower compared with the IAI (SOD: 245.50 ± 8.41 NU/mg prot; GSH: 24.17 ± 1.29 nmol/mg prot, *n*=10), IAH (SOD: 232.40 ± 10.50 NU/mg prot; GSH: 24.73 ± 1.07 nmol/mg prot, *n*=9), and control groups (SOD: 550.60 ± 21.84 NU/mg prot; GSH: 36.81 ± 2.15 nmol/mg prot, *n*=12).

## Discussion

IAI and IAH are critical problems in clinical practice. Damage of intestinal mucosal barrier function is the trigger and a major factor of MODS [5,9]. Therefore, it is necessary to diagnose and treat the damage in a timely way [10]. We successfully established an animal model of IAI [11] and IAH [8,12]. Previous studies [12–16] only focussed on the impact of a single factor (IAI or IAH) in their corresponding animal models. To date, there has been no report of a combined IAI and IAH animal model.

Here, we report the establishment of a rabbit model that simulates IAI combined with IAH, and verify its effectiveness by evaluating damage of intestinal mucosal barrier function. The study also compared intestinal mucosal barrier dysfunction amongst four groups: IAI combined with IAH, IAI alone, IAH alone, and healthy controls. We report that the combined effect of IAI and IAH aggravated tissue damage in the model, which was significantly more severe than the single factor groups.

The mortality of the IAI and IAH group was significantly higher amongst the groups. The effect was similar to that seen in clinical practice, as IAI combined with IAH is a life-threatening condition [17].

In addition to the mortality rate, we performed pathologic analyses. Severe damage to the intestinal mucosa in the IAI and IAH group was observed under light microscopy. As expected, histopathologic scores were also highest in the IAI and IAH group compared with the other groups, which was consistent with the light microscopy findings. Obvious structural damage of the tight junctions was observed by TEM in the IAI and IAH group.

In the IAI and IAH rabbit model, the plasma concentration of FITC-dextran was significantly higher compared with the other groups. FITC-dextran [18] is a commonly used method for testing intestinal barrier permeability. The intestinal barrier permeability was seriously impaired in the IAI and IAH group.

A similar trend was evident for the studies using LPS. LPS is released by breakdown of the cell walls of Gram-negative bacteria, and is an index to measure intestinal barrier permeability [19]. Trends of FITC-dextran and LPS were different amongst the four groups. A large amount of LPS was released into the blood after intestinal mucosal barrier damage under stress states (including IAI and IAH). This might explain the inconsistencies in data between FITC-dextran and LPS. Changes in microbial composition might also be related to these inconsistencies. Leng et al. [12] found that in an IAH model, a Gram-positive bacteria, Firmicutes, was significantly decreased, while a Gram-negative bacteria, Proteobacteria, was increased markedly. Hence, increased LPS was released into the blood in the IAH model. Further research is required to explore this hypothesis. Following intestinal mucosal barrier damage, LPS was released into the blood circulation, which can cause sepsis, MODS, or even death [19]. Therefore, an early diagnosis and timely intervention are critical to prevent adverse outcomes.

d-Lactate is a bacterial metabolite produced by several kinds of intestinal bacteria. Under stress status, bacteria multiply and in addition to an increase in intestinal mucosal permeability, might result in increased concentrations of d-Lactate released into the blood [20]. Therefore, monitoring the plasma concentration of d-Lactate might reflect intestinal mucosal damage and permeability. Similar to the LPS study, the plasma concentration of d-Lactate was highest in the IAI and IAH group, indicating that the intestinal mucosal barrier function was markedly altered.

When severe infection or even sepsis occurs, the levels of inflammatory mediators increase, leading to inflammatory cascade reactions and damage to villus cells. DAO is an enzyme with high activity in villus cells, especially in the ileal mucosa tissue. Therefore, increased concentrations of DAO in ileal mucosa tissues suggests intestinal barrier damage [21]. Unsurprisingly, in our study, the concentration of DAO was highest in the IAI and IAH group compared with the other groups.

The structures of tight junctions were impaired in the experimental groups, especially in the IAI and IAH group. However, the expression of tight junction protein family remained unclear in the rabbit model. Occludin [22–24], a tight junction protein, is a representative marker of intestinal mucosal barrier dysfunction. The expressions of occludin protein and mRNA in ileum mucosa tissue were low in the IAI and IAH group. These results confirmed the findings by SEM, and revealed the molecular mechanisms involved in the IAI and IAH group.

However, occludin expression shown in Figure 4 does not correlate with the data in Figure 3, suggesting that there might be additional factors that contribute to damage in the IAI and IAH model. After measuring the protein and mRNA levels of other tight junction proteins (Claudin-2, Claudin-5, JAM-A, and ZO-1), no significant differences were found between the groups. This suggests factors other than these are involved in the IAH and IAI model, which we will explore in the future.

MDA, a representative oxidizing substance in ileum mucosa tissues, was used to evaluate the oxidizing status. The concentration of MDA was highest in IAI and IAH group compared with the other groups. In contrast, SOD and GSH, representative reducing substances in ileum mucosa tissues, were lowest in the IAI and IAH group. Leng et al. [25] found that IAH caused adverse effects based on the pro-oxidant–antioxidant balance. In our study, the levels of MDA were increased significantly, whereas those of the antioxidant substances, GSH-Px and SOD tended to decline. Given the above, the intestinal mucosa tissue in the IAI and IAH group indicated oxidizing stress status.

There were some limitations in the present study. This study established an animal model that was used to observe pathologic changes. However, the underlying mechanisms of IAI combined with IAH are still unclear. Previous studies [12,25,26] using simple IAH models reported that intestinal bacterial translocation and Toll-like receptor 4 were the underlying mechanisms of IAH-derived sepsis. These factors might also be involved in the IAI combined with IAH model, where other signal pathways participate in the pathologic changes. Further research has been done by our team to validate the mechanisms. Strier et al. [26] found that in the IAH group (IAP at 15 mm Hg), high amounts of bacteria (such as *Escherichia coli, Enterococcus faecalis, Staphylococcus*, and *Streptococcus*) translocated to the mesenteric lymph nodes, portal vein blood, and liver. Leng et al. [12] reported changes in the microbial composition during IAH (IAP at 20 mm Hg). Furthermore, IAH resulted in a decrease in the relative abundance of Firmicutes species and an increase in the relative abundance of Proteobacteria. Thus, disturbed host–microbiota interactions might result in gut-derived sepsis.

Changes in microbial composition were also reported in IAI patients [27]. Therefore, changes in microbial composition are of great research interest. The use of probiotics might be a new clinical treatment for critically ill patients that develop IAI and IAH.

Further research is needed to explore the pathophysiologic foundations and underlying mechanisms of IAI combined with IAH. Better therapies and interventions might be applied in clinical practice by means of the animal model established here.

## Conclusion

IAI combined with IAH aggravates damage of intestinal mucosa tissue in a rabbit model, as well as altering intestinal mucosa barrier functions (especially the tight junction protein, occludin) and intestinal mucosa redox status. The combined effects were significantly more severe than a single factor alone. IAI combined with IAH should be prevented and treated effectively in clinical practice.

## Abbreviations

CLP: cecal ligation puncture
DAO: diamine oxidase
FITC-dextran: FITC-conjugated dextran
IAH: intra-abdominal hypertension
IAI: intra-abdominal infection
IAP: intra-abdominal pressure
MDA: malonaldehyde
MODS: multiple organ dysfunction syndrome
real-time PCR: quantitative real-time PCR
SOD: superoxide dismutase

## References

[1] Vincent J. L., Rello J., Marshall J., Silva E., Anzueto A., Martin C.D. (2009) International Study of the Prevalence and Outcomes of Infection in Intensive Care Units. JAMA 302, 2323–2329 10.1001/jama.2009.1754 19952319

[2] Mazuski J.E., Tessier J.M., May A.K., Sawyer R.G., Nadler E.P., Rosengart M.R. (2017) The Surgical Infection Society Revised Guidelines on the Management of Intra-Abdominal Infection. Surg. Infect. (Larchmt.) 18, 1–76 10.1089/sur.2016.261 28085573

[3] Kirkpatrick A. W., Roberts D.J., De Waele J., Jaeschke R., Malbrain M., De Keulenaer B. (2013) Intra-abdominal hypertension and the abdominal compartment syndrome: updated consensus definitions and clinical practice guidelines from the World Society of the Abdominal Compartment Syndrome. Intensive Care Med. 39, 1190–1206 10.1007/s00134-013-2906-z 23673399 PMC3680657

[4] Kim I.B., Prowle J., Baldwin I. and Bellomo R. (2012) Incidence, risk factors and outcome associations of intra-abdominal hypertension in critically ill patients. Anaesth. Intensive Care 40, 79–89 22313065 10.1177/0310057X1204000107

[5] Mittal R. and Coopersmith C.M. (2014) Redefining the gut as the motor of critical illness. Trends Mol. Med. 20, 214–223 10.1016/j.molmed.2013.08.004 24055446 PMC3959633

[6] Piton G. and Capellier G. (2016) Biomarkers of gut barrier failure in the ICU. Curr. Opin. Crit. Care 22, 152–160 26808138 10.1097/MCC.0000000000000283

[7] Huang Y., Wang S.R., Yi C., Ying M.Y., Lin Y. and Zhi M.H. (2002) Effects of recombinant human growth hormone on rat septic shock with intraabdominal infection by E. coli. World J. Gastroenterol. 8, 1134–1137 10.3748/wjg.v8.i6.1134 12439940 PMC4656395

[8] Cheng J., Wei Z., Liu X., Li X., Yuan Z., Zheng J. (2013) The role of intestinal mucosa injury induced by intra-abdominal hypertension in the development of abdominal compartment syndrome and multiple organ dysfunction syndrome. Crit. Care 17, R283 10.1186/cc13146 24321230 PMC4057115

[9] Doig C.J., Sutherland L.R., Sandham J.D., Fick G.H., Verhoef M. and Meddings J.B. (1998) Increased intestinal permeability is associated with the development of multiple organ dysfunction syndrome in critically ill ICU patients. Am. J. Res. Crit. Care Med. 158, 444–451 10.1164/ajrccm.158.2.9710092 9700119

[10] Vidal M.G., Weisser J.R., Gonzalez F., Toro M.A., Loudet C., Balasini C. (2008) Incidence and clinical effects of intra-abdominal hypertension in critically ill patients. Crit. Care Med. 36, 1823–1831 10.1097/CCM.0b013e31817c7a4d 18520642

[11] Toscano M.G., Ganea D. and Gamero A.M. (2011) Cecal ligation puncture procedure. J. Vis. Exp. 10.3791/2860 PMC3339843 21587163

[12] Leng Y.X., Yi M., Fan J., Bai Y., Ge Q.G. and Yao G.Q. (2016) Effects of acute intra-abdominal hypertension on multiple intestinal barrier functions in rats. Sci. Rep. 6, 22814 10.1038/srep22814 26980423 PMC4793228

[13] Swidsinski A., Loening-Baucke V., Theissig F., Engelhardt H., Bengmark S., Koch S. (2007) Comparative study of the intestinal mucus barrier in normal and inflamed colon. Gut 56, 343–350 10.1136/gut.2006.098160 16908512 PMC1856798

[14] Li G.-x., Wang X.-m., Jiang T., Gong J.-f., Niu L.-y. and Li N. (2014) Berberine prevents damage to the intestinal mucosal barrier during early phase of sepsis in rat through mechanisms independent of the NOD-like receptors signaling pathway. Eur. J. Pharm. 730, 1–7 10.1016/j.ejphar.2014.02.006 24530556

[15] Short S.S., Wang J., Castle S.L., Fernandez G.E., Smiley N., Zobel M. (2013) Low doses of Celecoxib attenuate gut barrier failure during experimental peritonitis. Lab. Invest. 93, 1265–1275 10.1038/labinvest.2013.119 24126890 PMC3966546

[16] Al-Bahrani A.Z., Darwish A., Hamza N., Benson J., Eddleston J. M., Snider R.H. (2010) Gut barrier dysfunction in critically ill surgical patients with abdominal compartment syndrome. Pancreas 39, 1064–1069 10.1097/MPA.0b013e3181da8d51 20861696

[17] Soop M. and Carlson G. L. (2017) Recent developments in the surgical management of complex intra-abdominal infection. Br. J. Surg. 104, E65–E74 10.1002/bjs.10437 28121035

[18] Nejdfors P., Ekelund M., Jeppsson B. and Westrom B. R. (2000) Mucosal *in vitro* permeability in the intestinal tract of the pig, the rat, and man: Species- and region-related differences. Scand. J. Gastroenterol. 35, 501–507 10.1080/003655200750023769 10868453

[19] Ewaschuk J., Endersby R., Thiel D., Diaz H., Backer J., Ma M. (2007) Problotic bacteria prevent hepatic damage and maintain colonic barrier function in a mouse model of sepsis. Hepatology 46, 841–850 10.1002/hep.21750 17659579

[20] Wu G.H., Wang H., Zhang Y.W., Wu Z.H. and Wu Z.G. (2004) Glutamine supplemented parenteral nutrition prevents intestinal ischemia-reperfusion injury in rats. World J. Gastroenterol. 10, 2592–2594 10.3748/wjg.v10.i17.2592 15300914 PMC4572171

[21] Zhang C., Sheng Z.Y., Hu S., Gao J.C., Yu S. and Liu Y. (2002) The influence of apoptosis of mucosal epithelial cells on intestinal barrier integrity after scald in rats. Burns 28, 731–737 10.1016/S0305-4179(02)00210-3 12464470

[22] Furuse M., Hirase T., Itoh M., Nagafuchi A., Yonemura S., Tsukita S. (1993) Occludin - a novel integral membrane-protein localizing at tight junctions. J. Cell. Biol. 123, 1777–1788 10.1083/jcb.123.6.1777 8276896 PMC2290891

[23] Hirase T., Staddon J. M., Saitou M., AndoAkatsuka Y., Itoh M., Furuse M. (1997) Occludin as a possible determinant of tight junction permeability in endothelial cells. J. Cell Sci. 110, 1603–1613 9247194 10.1242/jcs.110.14.1603

[24] McCarthy K. M., Skare I. B., Stankewich M. C., Furuse M., Tsukita S., Rogers R. A. (1996) Occludin is a functional component of the tight junction. J. Cell Sci. 109, 2287–2298 8886979 10.1242/jcs.109.9.2287

[25] Leng Y., Zhang K., Fan J., Yi M., Ge Q., Chen L. (2014) Effect of acute, slightly increased intra-abdominal pressure on intestinal permeability and oxidative stress in a rat model. PLoS ONE 9, e109350 10.1371/journal.pone.0109350 25295715 PMC4190173

[26] Strier A., Kravarusic D., Coran A. G., Srugo I., Bitterman N., Dorfman T. (2017) The effect of elevated intra-abdominal pressure on TLR4 Signaling in intestinal mucosa and on intestinal bacterial translocation in a rat. J. Laparoendosc. Adv. Surg. Tech. A 27, 211–216 10.1089/lap.2016.0212 27875107

[27] de Ruiter J., Weel J., Manusama E., Kingma W. P. and van der Voort P. H. (2009) The epidemiology of intra-abdominal flora in critically ill patients with secondary and tertiary abdominal sepsis. Infection 37, 522–527 10.1007/s15010-009-8249-6 19669089

